# Parental safety concerns and active school commute: correlates across multiple domains in the home-to-school journey

**DOI:** 10.1186/1479-5868-11-32

**Published:** 2014-03-06

**Authors:** Abiodun O Oluyomi, Chanam Lee, Eileen Nehme, Diane Dowdy, Marcia G Ory, Deanna M Hoelscher

**Affiliations:** 1 The Michael & Susan Dell Center for Healthy Living, UT School of Public Health, Austin Regional Campus, The University of Texas Health Science Center at Houston, 1616 Guadalupe, Suite 6.300, Austin, TX 78701, USA; 2 Department of Landscape Architecture and Urban Planning, College of Architecture, Texas A&M University, W014D Williams Administration Building, College Station, TX 77843-3137, USA; 3 School of Rural Public Health, Texas A&M Health Science Center, SRPH Administration Building, College Station, TX 77843-1266, USA

**Keywords:** Active commuting to school, Walking to school, Child pedestrian, Traffic safety, Personal safety, Crime safety, Pedestrian safety, Physical activity, Environmental perception, Safe routes to school

## Abstract

**Background:**

Empirical evidence of the relationship between safety concerns and walking to school (WTS) is growing. However, current research offers limited understanding of the multiple domains of parental safety concerns and the specific mechanisms through which parents articulate safety concerns about WTS. A more detailed understanding is needed to inform environmental and policy interventions. This study examined the relationships between both traffic safety and personal safety concerns and WTS in the U.S.

**Methods:**

This cross-sectional analysis examined data from the Texas Childhood Obesity Prevention Policy Evaluation (T-COPPE) project, an evaluation of state-wide obesity prevention policy interventions. All study data were from the survey (n = 830) of parents with 4th grade students attending 81 elementary schools across Texas, and living within two miles from their children's schools. Traffic safety and personal safety concerns were captured for the home neighborhood, en-route to school, and school environments. Binary logistic regression analysis was used to assess the odds of WTS controlling for significant covariates.

**Results:**

Overall, 18% of parents reported that their child walked to school on most days of the week. For traffic safety, students were more likely to walk to school if their parent reported favorable perceptions about the following items in the home neighborhood environment: higher sidewalk availability, well maintained sidewalks and safe road crossings. For the route to school, the odds of WTS were higher for those who reported "no problem" with each one of the following: traffic speed, amount of traffic, sidewalks/pathways, intersection/crossing safety, and crossing guards, when compared to those that reported "always a problem". For personal safety in the en-route to school environment, the odds of WTS were lower when parents reported concerns about: stray or dangerous animals and availability of others with whom to walk.

**Conclusions:**

Findings offered insights into the specific issues that drive safety concerns for elementary school children’s WTS behaviors. The observed associations between more favorable perceptions of safety and WTS provide further justification for practical intervention strategies to reduce WTS barriers that can potentially bring long-term physical activity and health benefits to school-aged children.

## Background

The emerging attention focused on walking to school (WTS), particularly in industrialized countries, is grounded in the recognition of the importance of physical activity among children who are adopting increasingly sedentary lifestyles [1,2]. Physical activity has both a positive, direct effect on children’s health and an indirect effect through its role in healthy weight maintenance or weight loss among the overweight [3,4]. The effect of physical activity on adiposity makes it an essential component in combating the childhood obesity epidemic, and studies have documented a positive relationship between WTS and other forms of physical activity. Recent studies have shown that elementary school students who walk/bike to school (1) may obtain more daily physical activity than those using motorized commuting modes [5]–[8]; (2) are more likely to engage in physical activity outside school [6,9,10]; and (3) are more likely to walk/bike to other non-school destinations [11].

Despite its potential health benefits, rates of active commuting to school (e.g. walking and bicycling) have plummeted over the last four decades in the U.S. In 2009, only 12.7% of elementary and middle school students walked or biked to school compared with 47.7% in 1969 [12]. Several reasons for this sharp drop in active commuting to school (ACS) have been identified by parents with school-aged children (5-18 years old), including distance (62%), traffic-related danger (30.4%), weather (18.6%), crime (11.7%), and school policy (6.0%) [13]. For WTS, two of the most frequently reported barriers are long distance [14]–[18] and safety concerns [19]–[21]. Addressing the distance barrier, while being the most influential factor predicting the school travel model choice, is difficult as it requires multi-faceted environmental interventions involving policy changes in land use, school siting, attendance zone, etc. [22]. In comparison, environmental changes to alleviate safety barriers to WTS may be more readily implementable.

While safety concerns are hypothesized barriers to WTS, there is clearly the need for more focused empirical inquiries into the potential relationship between these two phenomena because current research offers little in terms of exploring/explaining the mechanisms through which safety concerns might impact active transport [23,24]. Generally, safety concerns have been investigated in terms of road safety (traffic- or pedestrian-related safety concerns) and personal safety (crime- or predator-related safety concerns). Better understanding of the relationships between multiple domains of safety concerns and WTS can contribute to the development of practical intervention strategies to reduce barriers to WTS, which may lead to increases in physical activity and long-term health benefits to school-aged children.

To contribute to the growing yet limited body of literature on safety and WTS, we examined the relationships between WTS and specific measures of road and personal safety measures in a sample of U.S. schoolchildren who were selected from elementary schools across Texas. We also examined the relationships between selected covariates and walking, in order to obtain insights into the relations between these covariates in our population, as well as to adjust for the effects of the socio-demographic covariates in the potential relationships between safety concerns and WTS.

## Methods

### Design

This was a cross-sectional study using the baseline parental survey data from the Texas Childhood Obesity Prevention Policy Evaluation (T-COPPE) project. T-COPPE is an ongoing 5-year project that evaluates state-level implementation of two key national obesity prevention policies in Texas: the Safe Routes to School (SRTS) program and the Women, Infants and Children (WIC) revised food package. T-COPPE aims to: (1) inform decision makers about the effectiveness of these policies, and (2) assist local, state, and national policymakers to identify policies for promoting children's healthy eating and increased physical activity. At baseline (2009), T-COPPE recruited a total of 81 schools to participate in the project from 58 cities in 43 counties where the Texas Department of Transportation had approved SRTS projects as part of SAFETEALU (Safe Accountable Flexible Efficient Transportation Equity Act: A Legacy for Users). All study protocols and instruments were approved by The University of Texas Health Science Center at Houston Institutional Review Board.

### Sample

All 4th grade students and their parents (n = 6,500 pairs) from the approved schools were invited to complete T-COPPE baseline surveys. A total of 2,053 (31.6%) parent surveys and 3,315 (51%) student surveys were returned, out of which were 1,635 parent-student dyads. About eighty percent (n = 1,305) of the dyads’ home addresses were successfully geocoded using a geographic information system (GIS) technology (ESRI, ArcGIS 10.0). The inclusion criterion for the current analysis was that the residential address of the participant must be within *walkable distance* to their school, as defined by living within a 2-mile (3.2 km) distance from their school. This distance was selected since, according to the State of Texas, a student must live two miles or more from his/her assigned school to be eligible for free regular education school bus transportation [25]. The two-mile distance was determined based on the objectively-measured, shortest network distance from home to school, using GIS. Out of the 857 living in the two-mile distance to school, the mode choice to school was not reported for 27, leaving 830 participants for analysis.

### Measures

The parents of students who participated in the T-COPPE study received a packet that consisted of the consent form and parent survey prior to the student survey administration, which occurred at the child’s school. Parents returned the consent and completed survey to their child’s teacher in a sealed envelope. For the outcome of interest, *walking to school* (WTS), we used the relevant question from the National Safe Route To School Survey. Children were classified as walkers if their parents answered “walking” to the question – “On most days how does your 4th grade child arrive at school and leave after school?” [26]. Since WTS has been shown to vary significantly by certain individual- and societal-level characteristics [27]–[29], we assessed selected covariates in terms of five themes: socio-demographic status, acculturation, medical condition, school policy, and social capital (civic engagement and social integration). The primary exposures of interest for the current study were *perceived traffic safety concerns* and *perceived personal safety concerns*, examined across three environmental domains in the home-to-school journey – the home neighborhood environment, the en-route to school environment, and the school environment.

All data analyzed in the current study were retrieved from the T-COPPE survey. Questions in the T-COPPE survey were adapted from several surveys, including: the National Center for Safe Routes To School Parent Survey [26]; the School Physical Activity and Nutrition (SPAN) parent survey [30]; the Urban Hispanic Perceptions of Environment and Activity Among Kids (UH-PEAK) [31]; the Neighborhood Environment Walkability Survey (NEWS) [32,33]; and the TV reduction intervention study (En Vivo) [34]. Additional questions were adapted from specific relevant published reports [35,36]. Questions used in the current study, their response types, and their sources are listed in Table 1.

**Table 1:** Variables, the response types used, and the sources of the questions used

**Variables**		**Response type**	**Sources**
**Traffic safety**			
Home neighborhood environment			
(1)	Availability and quality of sidewalks	Rating scale (Likert)	[32,33,37]
(2)	Safe road crossings	Rating scale (Likert)	[32,33,37]
(3)	Observance of other people walking or bicycling	Rating scale (Likert)	[32,33,37]
En-route environment			
(4)	Availability of sidewalks/pathways	Rating scale (Likert)	[31]
(5)	Safety at intersections/crossings	Rating scale (Likert)	[31]
(6)	Crossing guards; and the amount/speed of traffic	Rating scale (Likert)	[31]
School environment			
(7)	Availability and quality of sidewalks	Rating scale (Likert)	[31]
(8)	Availability and quality of bike lanes/paths and bike racks	Rating scale (Likert)	[31]
(9)	Trees along the streets; and safe road crossings.	Rating scale (Likert)	[31]
**Personal Safety**			
Home neighborhood environment			
(10)	Safety of their child to walk or bike	Rating scale (Likert)	[33,37]
(11)	Personal sense of fear when walking outside alone after dark	Rating scale (Likert)	[33,37]
En-route environment			
(12)	Availability of adults or other children to walk with	Rating scale (Likert)	[31]
(13)	Violence or crime (e.g. Bullying/gangs)	Rating scale (Likert)	[31]
(14)	And stray or dangerous animals	Rating scale (Likert)	[31]
School environment			
(15)	Attractive buildings or natural things to see	Rating scale (Likert)	[31]
(16)	Abandoned houses or vacant lots	Rating scale (Likert)	[31]
(17)	Condoms and drug-related paraphernalia	Rating scale (Likert)	[31]
(18)	Well-maintained homes/apartments and gardens	Rating scale (Likert)	[31]
(19)	Other people who walk/bike	Rating scale (Likert)	[31]
**Potential Covariates**			
Socio-demographic status			
(20)	Government public assistance	Binary response	[38,39]
(21)	Respondent’s highest level of education	Multiple options	[38,39]
(22)	The family car-ownership status	Multiple options	[40]
Acculturation			
(23)	The language parents spoke in “most of the time”	Multiple options	[41]
(24)	The language parents thought in “most of the time”	Multiple options	[41]
(25)	Parents reported if child's grandparents were born in the US	Binary response	[41]
(26)	Parents reported if they were born in the US	Binary response	[41]
(27)	Parents reported if their children were born in the US	Binary response	[41]
Medical condition			
(28)	Medical conditions that limit physical activity for parents	Binary response	[38,39]
(29)	Medical conditions that limit physical activity for child	Binary response	[38,39]
(30)	Child has asthma	Binary response	
(31)	If asthma, is it well controlled	Binary response	
School policy			
(32)	Teachers encouraged students to walk/bike to school	Binary response	
(33)	Schools had a walking school bus program	Binary response	
(34)	Child’s school encouraged or discouraged walk/bike to school	Rating scale (Likert)	
Social capital: civic engagement			
(35)	Voted in an election (local, state, or national)	Binary response	[35,36]
(36)	Written or called a government official about community issue	Binary response	[35,36]
(37)	Attended a meeting of any government body	Binary response	[35,36]
(38)	Volunteered at the child’s school	Binary response	[35,36]
(39)	Volunteered for any community organization	Binary response	[35,36]
Social capital: social integration			
(40)	People in my community work together to resolve problems	Rating scale (Likert)	[36]
(41)	People in my community are only out for themselves	Rating scale (Likert)	[36]
(42)	A small group of people has all the power in my community	Rating scale (Likert)	[36]
(43)	I feel like an outsider in my community	Rating scale (Likert)	[36]
(44)	There is nothing I can do to solve problems in my community	Rating scale (Likert)	[36]

### Analysis

All statistical analyses were conducted in SPSS version 19. We assessed the relationships between potential covariates and WTS with chi-square tests. For each theme, multiple (×) comparisons were performed to assess the association between each constituent variable in the theme and WTS. Therefore, the test specific Bonferroni alpha level significance adjustment for the chi-square tests of p ≤ (0.05/×) was used to conserve the family-wise error rate of 0.05. For example, the alpha level of significance was p ≤ 0.01 for the socio-demographic factors, since five different comparisons were performed. For exploratory purposes, we checked for correlations among the selected variables for each theme (demography/SES evaluated separately from school policy variables), using Spearman's Rho tests (ρ). We also examined multicollinearity using the variance inflation factor (VIF).

Second, bivariate analyses of each exposure variable (by environment; i.e. home neighborhood, en-route, and school environment) were conducted with the dichotomous outcome measure of WTS. Logistic regression models were used to determine unadjusted odds ratios. Next, we performed a series of multivariable regression models, controlling for certain socio-demographic/SES factors that we chose as potential confounding variables; i.e. student's ethnicity, any type of public assistance (family), car ownership (family). These were chosen based on the prior knowledge of their relationships with the outcome (WTS) and neighborhood of residence – which is expected to inform neighborhood perceptions. The Hosmer-Lemeshow goodness-of-fit statistic was used to assess model fit. Models that provided a good fit to the data had a small test statistic and a large p value (*p* > established cutoff of 0.05).

## Results

### Population characteristics and relationship with walking to school

Overall, 18.7% of parents reported WTS as their child’s commute mode choice while only 1.8% biked. The remaining 79.5% used a combination of transit, car-pooling, and family vehicle (Table not shown). Table 2 presents data on sample characteristics and their relationships with WTS. Boys and girls were equally represented in the study, with majority being Hispanics. Almost one-third of the families received public assistance, most parents reported high school or General Education Development Certificate (GED) as their highest level of education, and almost every family had a vehicle. The majority of the parents were born in the US, most of them thought and spoke in the English language, and a very small proportion of the students were born outside the US. In the exploratory analyses of study characteristics as covariates (Table 2), the following groups were more likely to have walked to school when compared to their counterparts, at the Bonferroni adjustment alpha level – *p ≤ (0.05/×)* – families that received any public assistance; students from families that owned no/one vehicle; students whose teachers encouraged active school commuting; and students whose parents reported that child’s school encouraged active commuting. Other covariates that showed significance at p ≤ 0.05 included: parent voting in election, attending civic meetings, or volunteering at child’s school.

**Table 2:** Population characteristics and their relationships with walking to school

	***Totals***	**All**		**Nonwalkers**		**Walkers**		**χ**^**2 **^**( *p *)**	
	***830***	**N**	**(%)**	**N = 675**	**(%)**	**N = 155**	**(%)**		
**Demography & SES** (Bonferroni alpha level = 0.01)									
**Student gender**^**‡**^	*830*								
Boy		412	(49.6)	328	(48.6)	84	(54.2)	*.208*	
Girl		418	(50.4)	347	(51.4)	71	(45.8)		
**Student race/ethnicity**^**‡**^	*826*								
Non-Hispanic Whites		165	(20.0)	145	(21.6)	20	(12.9)	*.061*	
Mexican-American Latino Hispanics		507	(61.4)	399	(59.5)	108	(69.7)		
African-Americans		53	(6.4)	45	(6.7)	8	(5.2)		
Others		101	(12.2)	82	(12.2)	19	(12.3)		
**Does family receive any public assistance?**	*777*								
No		244	(31.4)	213	(33.6)	31	(21.5)	*.005*	**
Yes		533	(68.6)	420	(66.4)	113	(78.0)		
**Highest level of education for self?**	*691*								
Up to middle school or less		119	(17.2)	94	(16.6)	25	(19.8)	*.301*	
High School or GED		364	(52.7)	294	(52.0)	70	(55.6)		
Associate degree to professional degree		208	(30.1)	177	(31.3)	31	(24.6)		
**Does family own car, van or truck?**	*801*								
No		30	(3.7)	18	(2.8)	12	(8.2)	*.004*	**
Yes, one		326	(40.7)	263	(40.2)	63	(42.9)		
Yes, two or more		445	(55.6)	373	(57.0)	72	(49.0)		
**Acculturation** (Bonferroni alpha level = 0.008)									
**Language spoken most of the time by parent**	*800*								
Spanish		179	(22.4)	143	(22.0)	36	(24.2)	*.807*	
English		474	(59.3)	389	(59.8)	85	(57.0)		
English + others		147	(18.4)	119	(18.3)	28	(18.8)		
**Language thought in most of the time by parent**	*818*								
Spanish		193	(23.6)	153	(23.0)	40	(26.3)	*.527*	
English		501	(61.2)	414	(62.2)	87	(57.2)		
English + others		124	(15.2)	99	(14.9)	25	(16.4)		
**Were you born in US?**	*767*								
No		228	(29.7)	179	(28.6)	49	(34.8)	*.148*	
Yes		539	(70.3)	447	(71.4)	92	(65.2)		
**Was your mother born in US?**	*756*								
No		326	(43.1)	263	(42.7)	63	(45.0)	*.619*	
Yes		430	(56.9)	353	(57.3)	77	(55.0)		
**Was your father born in US?**	*747*								
No		323	(43.2)	257	(42.2)	66	(47.8)	*.228*	
Yes		424	(56.8)	352	(57.8)	72	(52.2)		
**Was your child born in US?**	*808*								
No		62	(7.7)	51	(7.8)	11	(7.3)	*.842*	
Yes		746	(92.3)	606	(92.2)	140	(92.7)		
**Medical limitations** (Bonferroni alpha level = 0.02)									
**Medical condition/disability that limit child’s PA?**	*822*								
No		772	(93.9)	624	(93.6)	148	(95.5)	*.365*	
Yes		50	(6.1)	43	(6.4)	7	(4.5)		
**Does child have asthma?**	*814*								
No		733	(90.0)	593	(89.8)	140	(90.9)	*.692*	
Yes		81	(10.0)	67	(10.2)	14	(9.1)		
**If yes, is asthma well controlled by medication?**	*105*								
No		29	(27.6)	27	(30.0)	2	(13.3)	*.181*	
Yes		76	(72.4)	63	(70.0)	13	(86.7)		
**School policy** (Bonferroni alpha level = 0.02)									
**Have teacher encouraged walk/bike to school?**^**‡**^	*669*								
No		558	(83.4)	472	(85.2)	86	(74.8)	*.006*	**
Yes		111	(16.6)	82	(14.8)	29	(25.2)		
**School has a walking school bus program?**^**‡**^	*476*								
No		349	(73.3)	300	(75.0)	49	(64.5)	*.057*	
Yes		127	(26.7)	100	(25.0)	27	(35.5)		
**School encourage walking/biking to/from school**	*408*								
Does not encourage		301	(73.8)	252	(78.5)	49	(56.3)	*<.001*	**
Encourage		107	(26.2)	69	(21.0)	38	(43.7)		
**Civic engagement** (Bonferroni alpha level = 0.01)									
**In the past 12 months have you…**									
**Voted in an election**	*797*								
No		433	(54.3)	341	(52.4)	92	(63.0)	*.020*	*
Yes		364	(45.7)	310	(47.6)	54	(37.0)		
**Written/called govt. official about community issue**	*790*								
No		718	(90.9)	584	(90.5)	134	(92.4)	*.479*	
Yes		72	(9.1)	61	(9.5)	11	(7.6)		
**Attended school board, city, or other govt. meeting**	*788*								
No		681	(86.4)	549	(85.0)	132	(93.0)	*.012*	*
Yes		107	(13.6)	97	(15.0)	10	(7.0)		
**Volunteered at your child’s school?**	*792*								
No		576	(72.7)	461	(71.3)	115	(79.3)	*.049*	*
Yes		216	(27.3)	186	(28.7)	30	(20.7)		
**Volunteered for any community org?**	*791*								
No		584	(73.8)	473	(73.2)	111	(76.6)	*.409*	
Yes		207	(26.2)	173	(26.8)	34	(23.4)		
**Social Integration** (Bonferroni alpha level = 0.01)									
**In my community where I live…**									
**people work together to resolve problems**	*801*								
Disagree		140	(17.5)	115	(17.6)	25	(17.1)	*.119*	
Unsure		343	(42.8)	270	(41.2)	73	(50.0)		
Agree		318	(39.7)	270	(41.2)	48	(32.9)		
**People are only out for themselves**	*800*								
Disagree		268	(33.5)	229	(34.9)	39	(27.1)	*.168*	
Unsure		327	(40.9)	260	(39.6)	67	(46.5)		
**A small group of people have all the power**	*796*								
Disagree		436	(54.8)	358	(54.9)	78	(54.2)	*.831*	
Unsure		268	(33.7)	217	(33.3)	51	(35.4)		
Agree		92	(11.6)	77	(11.8)	15	(10.4)		
**I feel like an outsider**	*787*								
Disagree		573	(72.8)	476	(73.7)	97	(68.8)	*.070*	
Unsure		145	(18.4)	110	(17.0)	35	(24.8)		
Agree		69	(8.8)	60	(9.3)	9	(6.4)		
**Nothing I can do to solve problems that happen**	*793*								
Disagree		443	(55.9)	369	(56.9)	74	(51.4)	*.249*	
Unsure		249	(31.4)	203	(31.3)	46	(31.9)		
Agree		101	(12.7)	77	(11.9)	24	(16.7)		

Texas 4th grade students, 2008-2010.

**‡**Questions that were answered by the children (students). Otherwise, questions were answered by parents.

*p ≤ 0.05; **p ≤ Bonferroni adjustment alpha level

### Relationships among covariates

Generally, correlations among significant covariates were low across the themes that we examined (Table not shown). For socio-demographic theme, three correlation pairs (including ethnicity) were between *ρ* = 0.109 and 0.289 (all *p* < 0.01). Two of the three correlation pairs for school policy were significant (*p* < 0.05), with highest *ρ* = 0.163, while all three correlation pairs for civic engagement were significant (*p* < 0.05), with highest *ρ* = 0.272. Secondary assessment of possible multicollinearity using the VIF supported lack of significant correlations among selected covariates; the highest VIF score across all the selected variables was 1.11. Based on the observed *ρ* values for demography and SES (potential confounders), multivariable analyses that include these covariates would not be affected by multicollinearity.

### Unadjusted and adjusted relationships between perceived road safety and walking to school

In the *home neighborhood* environment, bivariate analysis showed that three out of the four items in this domain were statistically significant. The likelihood (odds ratio) of walking was greater for students whose parents reported that there were sidewalks on most of their neighborhood streets than for those who reported no sidewalks. Similarly, there was increased likelihood of walking among two groups of students when compared to their counterparts: those whose parents reported that neighborhood sidewalks were well maintained, and that there were safe road crossings in their neighborhood. In the *en-route* environment, all five items examined showed significant associations with WTS. These were: speed of traffic along route to school; amount of traffic along route to school; intersection safety; crossing problems; and availability of crossing guards. In the *school* environment, WTS was higher when parents reported sufficient sidewalks near their child’s school vs. no sidewalks, as well as reporting availability of safe crossings vs. no safe crossings. Details presented in Table 3.

**Table 3:** Relationships between traffic safety and walking to school

	**Unadjusted**						**Adjusted†**				
	**N = 830**	**OR**	**95% CI**			***p***	**N = 830**	**OR**	**95% CI**		***p***
**Traffic safety (home)**											
**Sidewalks on most of neighborhood streets**	824						754				
No		1.00	Ref.			*<.001*		1.00	Ref.		*<.001*
Yes, a few		1.83	1.12	-	2.99			**1.87**	**1.11**	**3.16**	
Yes, many		2.38	1.53	-	3.71			**2.69**	**1.66**	**4.35**	
**Sidewalks in neighborhood well maintained**	700						634				
No		1.00	Ref.			*.029*		1.00	Ref.		*.005*
Yes, a few		1.47	0.90	-	2.39			1.68	0.99	2.86	
Yes, many		1.88	1.17	-	3.02			**2.20**	**1.30**	**3.71**	
**Safe road crossings in your neighborhood**	774						708				
No		1.00	Ref.			*.001*		1.00	Ref.		*<.001*
Yes, a few		1.84	1.16	-	2.91			**1.95**	**1.19**	**3.19**	
Yes, many		2.50	1.51	-	4.13			**2.61**	**1.51**	**4.49**	
**People walk/bike in your neighborhood**	812						743				
No		1.00	Ref.			*.356*		1.00	Ref.		*.001*
Yes, a few		1.35	0.66	-	2.74			1.45	0.68	3.08	
Yes, many		1.62	0.78	-	3.35			1.82	0.83	4.00	
**Traffic safety (en-route)**											
Always a problem		1.00	Ref.			*<.001*		1.00	Ref.		*<.001*
Sometimes a problem		1.68	0.99	-	2.83			**1.84**	**1.03**	**3.28**	
Not a problem		2.69	1.64	-	4.42			**2.86**	**1.64**	**4.99**	
**Amount of traffic along route a problem**	800						732				
Always a problem		1.00	Ref.			*<.001*		1.00	Ref.		*<.001*
Sometimes a problem		2.40	1.41	-	4.11			**2.72**	**1.51**	**4.87**	
Not a problem		3.66	2.17	-	6.17			**3.87**	**2.19**	**6.86**	
**Sidewalks or pathways a problem**	795						728				
Always a problem		1.00	Ref.			*<.001*		1.00	Ref.		*<.001*
Sometimes a problem		1.58	0.85	-	2.95			1.62	0.84	3.12	
Not a problem		3.35	1.99	-	5.66			**3.38**	**1.94**	**5.89**	
**Safety at intersections & crossings a problem**	801						736				
Always a problem		1.00	Ref.			*<.001*		1.00	Ref.		*<.001*
Sometimes a problem		2.89	1.52	-	5.49			**2.65**	**1.37**	**5.11**	
Not a problem		5.27	2.85	-	9.74			**4.75**	**2.54**	**8.89**	
**Crossing guards a problem**	792						727				
Always a problem		1.00	Ref.			*<.001*		1.00	Ref.		*<.001*
Sometimes a problem		2.58	1.13	-	5.87			**2.41**	**1.04**	**5.62**	
Not a problem		5.17	2.45	-	10.89			**4.90**	**2.29**	**10.46**	
**Traffic safety (school)**											
**Sidewalks on streets near child’s school**	814						745				
No		1.00	Ref.			*.003*		1.00	Ref.		*<.001*
Yes, a few		1.84	1.07	-	3.16			**2.05**	**1.14**	**3.67**	
Yes, many		2.41	1.41	-	4.10			**3.07**	**1.71**	**5.50**	
**Sidewalks well maintained**	736						670				
No		1.00	Ref.			*.086*		1.00	Ref.		*<.001*
Yes, a few		0.89	0.52	-	1.52			1.17	0.65	2.09	
Yes, many		1.39	0.83	-	2.34			**1.88**	**1.06**	**3.35**	
**Trees along streets near school**	796						727				
No		1.00	Ref.			*.201*		1.00	Ref.		*.001*
Yes, a few		1.52	0.90	-	2.56			**1.80**	**1.02**	**3.17**	
Yes, many		1.60	0.91	-	2.79			**2.07**	**1.12**	**3.82**	
**Bike lanes/paths or trails near school**	788						719				
No		1.00	Ref.			*.061*		1.00	Ref.		*<.001*
Yes, a few		1.56	1.04	-	2.36			**1.75**	**1.13**	**2.70**	
Yes, many		1.59	0.87	-	2.91			1.46	0.74	2.86	
**Bike lanes/paths or trails well maintained**	563						510				
No		1.00	Ref.			*.541*		1.00	Ref.		*.044*
Yes, a few		1.20	0.75	-	1.92			1.32	0.80	2.17	
Yes, many		1.34	0.76	-	2.37			1.43	0.76	2.68	
**Bike racks at or near school**	763						699				
No		1.00	Ref.			*.662*		1.00	Ref.		*.004*
Yes, a few		1.16	0.77	-	1.74			1.38	0.89	2.14	
Yes, many		1.28	0.70	-	2.33			1.40	0.72	2.72	
**Safe road crossings**	802						734				
No		1.00	Ref.			*.047*		1.00	Ref.		*<.001*
Yes, a few		1.86	1.11	-	3.13			**2.15**	**1.22**	**3.78**	
Yes, many		1.56	0.85	-	2.85			**2.06**	**1.06**	**4.00**	

Regression Analyses - (Unadjusted and Adjusted Odds Ratios): Texas 4th grade students, 2008-2010.

^†^Adjusted for: ***Socio-demographic*** - student's ethnicity, any type of public assistance (family), car ownership (family).

**Boldface** type indicates there was a significant difference with the reference group at 95 percent confidence interval in the adjusted model.

Each safety variable from the unadjusted bivariate analyses was included in a multivariable logistic model that included the selected confounders – student ethnicity, public assistance, and car ownership. The results are displayed in Table 3. For *home neighborhood* environments, the likelihood of WTS remained higher with higher sidewalk availability, well maintained sidewalks, and safe road crossings. Similarly, all items in the *en-route* environments retained significant relationships with WTS after adjustment. For the *school* environment, sidewalk on streets, bike lanes/paths, and safe road crossings maintained associations with WTS in the adjusted analyses. Sidewalk maintenance near school and trees along streets near school showed significant associations with WTS after adjusting for confounders. The Hosmer-Lemeshow (H-L) test indicated a good fit for each one of these multivariable models (data not shown).

### Unadjusted and adjusted relationships between perceived personal safety and walking to school

In the *home neighborhood* environment, bivariate analysis showed that one out of the four items in this domain was associated with WTS; parents who reported that it was safe for their child to walk or bike in the neighborhood also reported higher WTS when compared to their counterparts. In the *en-route* environment, children were less likely to report WTS if their parents reported some measure of concern on the following issues: having other adults or children to walk with; violence or crime problems; and stray or dangerous animals. None of the constituent variables for personal safety in the *school* environment showed significant association with WTS. More details are given in Table 4.

**Table 4:** Relationships between personal safety and walking to school

	**Crude**						**Adjusted†**					
	**N = 830**	**OR**	**95% CI**			***P***	**N = 830**	**OR**	**95% CI**			***p***
**Personal safety (home)**												
**Do you feel safe walking in neighborhood**^**‡**^	824						754					
Never		1.00	Ref.			*.986*		1.00	Ref.			*.003*
Some of the time		1.03	0.59	-	1.83			1.23	0.66	-	2.29	
Most/all of the time		1.04	0.64	-	1.72			1.42	0.82	-	2.47	
**Do you feel safe riding a bike in neighborhood**^**‡**^	826						756					
Never		1.00	Ref.			*.777*		1.00	Ref.			*.009*
Some of the time		0.86	0.48	-	1.53			0.90	0.49	-	1.67	
Most/all of the time		0.84	0.52	-	1.36			0.98	0.58	-	1.64	
**Safe for child to walk/bike in neighborhood**	802						737					
Never/not very often		1.00	Ref.			*.007*		1.00	Ref.			*< .001*
Some of the time		1.41	0.86	-	2.31			1.61	0.95	-	2.74	
Most/all of the time		2.01	1.28		3.17			**2.42**	**1.47**	**-**	**3.99**	
**Afraid when out alone after dark in community**	792						730					
Disagree		1.00	Ref.			*.643*		1.00	Ref.			*015*
Unsure		0.79	0.48	-	1.32			0.78	0.45	-	1.32	
Agree		0.99	0.66		1.50			1.03	0.67	-	1.60	
**Personal Safety (En-route)**												
**Adults, other children to walk/bike with**	785						720					
Not a problem		1.00	Ref.			*<.001*		1.00	Ref.			*<.001*
Sometimes a problem		0.46	0.29	-	0.74			**0.49**	**0.30**	**-**	**0.79**	
Always a problem		0.17	0.09	-	0.34			**0.16**	**0.08**	**-**	**0.33**	
**Violence or crime a problem**	799						732					
Not a problem		1.00	Ref.			*.010*		1.00	Ref.			*<.001*
Sometimes a problem		0.60	0.39	-	0.93			**0.56**	**0.35**	**-**	**0.89**	
Always a problem		0.44	0.20	-	0.94			**0.46**	**0.21**	**-**	**0.99**	
**Stray or dangerous animals a problem**	808						740					
Not a problem		1.00	Ref.			*<.001*		1.00	Ref.			*<.001*
Sometimes a problem		0.40	0.26	-	0.62			**0.42**	**0.27**	**-**	**0.66**	
Always a problem		0.75	0.39	-	1.46			0.69	0.34	-	1.39	
**Personal safety (school)**												
**Near child’s school…**												
**Attractive buildings and natural things to see**												
No	819	1.00	Ref.			*.708*	749	1.00	Ref.			*.002*
Yes, a few		0.86	0.59	-	1.25			1.05	0.70	-	1.58	
Yes, many		1.02	0.52	-	2.01			1.71	0.82	-	3.56	
**Abandoned houses or vacant lots**												
No	822	1.00	Ref.			*.947*	752	1.00	Ref.			*.005*
Yes, a few		1.06	0.74	-	1.53			1.03	0.69	-	1.51	
Yes, many		1.02	0.49	-	2.12			0.69	0.29	-	1.63	
**Condoms, drug-related paraphernalia (needles, syringes, etc.)**	809						741					
No		1.00	Ref.			*.704*		1.00	Ref.			*.008*
Yes, a few		0.73	0.32	-	1.65			0.88	0.38	-	2.06	
Yes, many		1.25	0.26	-	6.07			1.63	0.32	-	8.33	
**Well-maintained homes, apartments & gardens**	817						748					
No		1.00	Ref.			*.799*		1.00	Ref.			*.005*
Yes, a few		0.82	0.43	-	1.55			0.88	0.44	-	1.75	
Yes, many		0.80	0.41	-	1.54			0.97	0.47	-	1.99	

Regression Analyses - (Crude and Adjusted Odds Ratios): Texas 4th grade students, 2008-2010.

^†^Adjusted for: ***Socio-demographic*** - student's ethnicity, any type of public assistance (family), car ownership (family).

**‡**Questions that were answered by the children (students). Otherwise, questions were answered by parents.

**Boldface** type indicates there was a significant difference with the reference group at 95 percent confidence interval in the adjusted model.

Each personal safety variable was included in multivariable logistic regression models that adjusted for the selected confounders – student ethnicity, public assistance, and car ownership (Table 4). For *home neighborhood* environment, the likelihood of walking remained higher only among those who reported that it was safe for their child to walk or bike in the neighborhood. In the *en-route* environment, all significant associations that were observed in the unadjusted models remained after adjusting for confounders. The Hosmer-Lemeshow (H-L) tests indicated that all multivariable models fit reasonably well (data not shown).

## Discussion

In this cross-sectional study, we examined the associations of parental concerns related to safety on walking to school among 4th grade students who lived within a 2-mile network buffer of selected elementary schools across the state of Texas. A series of single-factor regression analyses were conducted to investigate safety concerns (road safety and personal safety) across three spatial domains (home neighborhood, en-route to school, and near the school). These analyses showed that, in general, children’s walking to school depended on parental perceptions of the following factors related to road safety: sidewalks and safe road crossings in the neighborhood; sidewalks, speed and amount of traffic, and intersections along school route; and sidewalks, crossing guards, and availability of trees along streets near the school. In terms of personal safety, parents were concerned about general neighborhood safety, stray or dangerous animals, and availability of adults with whom their child can walk en-route.

Our findings expand upon prior studies that suggest that parental safety concerns are related to walking to school among children. For instance, parental perception of the presence of sidewalks was found to be associated with walking to school in all three spatial domains studied. Two prior studies using children’s perspectives of the neighborhood did not find a significant association between the presence of sidewalks and walking to school [42,43]. Of three studies that used parent perceptions, two found a significant association [44,45] while one did not [46]. The parent’s perception of sidewalk availability may be more influential on children’s walking to school than the perception of the child. This may be particularly true for younger children; Trapp and colleagues studied children in grades 5-7 [46], while the students in the current study were in grade 4.

In the current study, we found more consistent associations between WTS and the road safety factors than the personal safety factors examined. The potential salience of road safety is highlighted when observed relationships with WTS are assessed in the *home* and *school* spatial domains. In the *home neighborhood* environment, three in four road safety items maintained significant relationships with WTS in the adjusted models, while one in four stayed significant for personal safety. A similar trend was observed for adjusted models in the *school* domain, with three in eight for traffic safety and zero in five for personal safety. This finding is in line with a nationally-representative study that found a greater proportion of parents felt that it was too dangerous for their 5-11 year old child to walk to school because of traffic than because of crime (37.0% vs. 14.2%) [47], as well as a prior review on attributes of the physical environment and children’s physical activity levels [48][37]. In this review, parental concerns about road hazards (street crossings and traffic) were more consistently associated with children’s physical activity levels than were perceptions of safety from crime.

Our findings suggest that the *en-route* environment may be the most critical environment to parents for both traffic safety and personal safety. All but one of the 8 items that were assessed in the *en-route* environment maintained significant relationships with walking to school in the expected direction, i.e. more safety concern associated with less walking to school. Comparatively, 4 of 8 and 3 of 13 items remained significant in adjusted models at the *home neighborhood* and *school* environment respectively. Further, the largest measures of effect were seen in the *en-route* domain. These findings suggest that parents may weigh the safety of the specific route a child will travel over the safety of the neighborhood or school environment when deciding whether to allow their child to walk to school. This finding lends further support to the call for specificity when defining the spatial domain of a behavior of interest [49].

Our assessment of the relationships between the selected covariates and WTS confirmed previous findings in some cases, and offered some additional insights. We saw a negative relationship between indicators of socio-economic status and walking to school, as has been generally, but not consistently, noted in other studies. A 2009 systematic review of determinants of children’s active travel reported negative associations with children’s active travel in six of seven studies that considered household income, nine of twelve studies considering car ownership, and four of twelve that considered parental education [24]. We also found that student perception of teacher support and parent perception of school support for active commuting had a positive association with students’ walking to school. A similar finding has been reported in at least one prior study [46]. Considering the low prevalence of this perception among students (16.6%) and parents (26.2%) in this study, school policy may be a practical target for interventions. For instance, schools may consider adopting an official policy statement to support active commuting to school and making this statement of support known to all families and the larger community. Also, we saw negative associations between several measures of civic engagement (voted in an election, attended a school board meeting, and volunteered in child’s school) and WTS, significant at the p < 0.05 level, although these were not significant with the Bonferroni correction. Taken together, these results suggest that children from higher SES families and those who are civically-engaged may be less likely to walk to school than their counterparts. Any relationships between these variables are likely complex, but do suggest that social norms may be involved. Further work in this area may be warranted.

Several potential limitations can be noted. The cross-sectional design precludes causal inference, and our findings were based on self-reported information, which may lead to recall bias. Respondent burden might have played some role in the general response rate of the parents (31.6%), and possibly influenced the reliability of reported study variables. However, other researchers and governmental organizations rely on self-reported information for their analyses, and evidence of a systematic bias due to self-reporting of mode choice to school is largely absent in the literature. Importantly, given that perceived safety was the primary exposure of interest in the current set of analyses, the use of survey was therefore an appropriate means of measuring participants’ perceptions. Another issue that is related to the assessment of perceived safety concerns and WTS is the potential for a mismatch between perceptions of safety and “actual safety”. Others have reported differing findings on the concordance between environmental perceptions and objective measures [50]–[52]. Therefore, if safety perceptions do not correspond well to actual risk in the home-to-school journey, attempts to improve traffic or personal safety “*on the ground*” might not increase WTS. Essentially, it may be that it is the perceptions of risk that need changing as much, or even more, than the actual environment. This point is being highlighted in the current paper, as an important theme in this subfield. Nonetheless, an in-depth critique is beyond the scope of the current study.

Despite the acknowledged limitations, our findings have relevance to the behavioral medicine field in a variety of ways. First of all, the current study asked participants about specific safety concerns, rather than using general safety questions, which provides evidence that road safety may be more relevant than personal safety to parents, as far as walking to school is concerned. However, despite this more robust assessment, the full range of parental perceptions around safety for their child may not be fully captured. Future research would benefit from the use of qualitative data gathering in communities (e.g. focus group discussions and interviews) to improve the operationalization of safety concern constructs. Secondly, a major contribution to existing knowledge is the level of spatial specificity offered by T-COPPE data that previous studies have lacked. This study provides the ability to examine relevant safety concerns across different spatial domains (i.e., *home neighborhood, en-route,* and *school* environments) going beyond previous single domain studies. Consequently, we were able to examine the differential effects in the exposure-outcome relationships across these spatially-distinct domains.

There are other prominent aspects of the T-COPPE study. T-COPPE participants were selected from both urban and rural schools across Texas; therefore, our findings may be generally applicable to Texas 4th grade students and their parents. Notably, since the current analyses included participants that live within a 2-mile distance from their school, this inclusion criterion addressed potential rural-urban distance-based differences. The T-COPPE population was more diverse and low income than previously reported data, and our sample is fairly large when compared to other similar studies. The methods used for construct development, data sourcing, and analyses can be replicated in most, if not all, settings.

## Conclusions

Results indicate that specific safety concerns in the neighborhood socio-environmental characteristics explained some of the variance in walking to school among 4th grade students in the present study. Of particular importance to parents is traffic safety along the route to the school. Based on these findings, we expect that increased focus on, and investment in, pedestrian-centric transportation infrastructures would result in increased WTS. In environments where active commuting to school has adequate infrastructure support, school encouragement of active commuting is recommended. In addition to enabling overall physical activity in children, such investments could result in a long-term population-wide health benefit, affecting all the people in the target neighborhoods.

## Competing interests

The authors declare that they have no competing interests.

## Authors’ contributions

AOO conceptualized and designed the study, assembled and processed the data for current analyses, oversaw the data analyses and the interpretations of findings, and led the overall writing of the article. CL conceptualized and designed the study, assembled and processed the data for current analyses, contributed to data analyses and interpretations, contributed to the drafting of the article. EKN was involved in data analyses and interpretations and contributed to the drafting of the article. DD, MO, and DMH supervised the data collection, access sharing and management for the parent study (T-COPPE) and contributed equally to the interpretation of findings. All authors edited drafts of the article for important intellectual content. All authors read and approved the final manuscript.
